# How does Nogo receptor influence demyelination and remyelination in the context of multiple sclerosis?

**DOI:** 10.3389/fncel.2023.1197492

**Published:** 2023-06-08

**Authors:** Zahra Rashidbenam, Ezgi Ozturk, Maurice Pagnin, Paschalis Theotokis, Nikolaos Grigoriadis, Steven Petratos

**Affiliations:** ^1^Department of Neuroscience, Central Clinical School, Monash University, Melbourne, VIC, Australia; ^2^Laboratory of Experimental Neurology and Neuroimmunology, Department of Neurology, AHEPA University Hospital, Thessaloniki, Greece

**Keywords:** myelin debris, Nogo-A, Nogo receptor 1, Nogo receptor 1-dependent axonopathy, oligodendrocyte, progressive multiple sclerosis, remyelination

## Abstract

Multiple sclerosis (MS) can progress with neurodegeneration as a consequence of chronic inflammatory mechanisms that drive neural cell loss and/or neuroaxonal dystrophy in the central nervous system. Immune-mediated mechanisms can accumulate myelin debris in the disease extracellular milieu during chronic-active demyelination that can limit neurorepair/plasticity and experimental evidence suggests that potentiated removal of myelin debris can promote neurorepair in models of MS. The myelin-associated inhibitory factors (MAIFs) are integral contributors to neurodegenerative processes in models of trauma and experimental MS-like disease that can be targeted to promote neurorepair. This review highlights the molecular and cellular mechanisms that drive neurodegeneration as a consequence of chronic-active inflammation and outlines plausible therapeutic approaches to antagonize the MAIFs during the evolution of neuroinflammatory lesions. Moreover, investigative lines for translation of targeted therapies against these myelin inhibitors are defined with an emphasis on the chief MAIF, Nogo-A, that may demonstrate clinical efficacy of neurorepair during progressive MS.

## 1. Introduction

Multiple sclerosis (MS) is defined as an inflammatory demyelinating and degenerative disorder which can affect the brain (Ruggieri et al., 2021), spinal cord (Moccia et al., 2020), and optic nerve (Mikolajczak et al., 2017) of adults with the mean age of onset over the last decade being 33 years (Rodríguez Murúa et al., 2022; Romero-Pinel et al., 2022). Even though the disease manifests in both males and females, it is more frequently diagnosed in women at the prime of their lives. Based on a nation-wide prevalence of MS study which was conducted in Sweden, it was reported by Ahlgren et al. (2011), that between 1960 and 2008 the prevalence of developing MS increased in women compared to men by a ratio of 2.3–3.5:1 (Ahlgren et al., 2011). Based on data extracted from the global MSBase registry (largest international registry of MS and neuroimmunological disorders) (Kalincik and Butzkueven, 2019), in February 2012, of the 18,885 registered patients from 25 countries whose data were recorded between 1951 and 2012, the frequency of annual relapse rates was 17.7% higher in females than males living with MS (Kalincik et al., 2013). Females dominate the incidence of relapsing-remitting MS (RRMS) as compared to their male counterparts by 2.3:1 ratio (Trojano et al., 2012; Sumelahti et al., 2014). However, in the primary progressive form of MS, males are affected twofold more than that of females (Luetic and Menichini, 2021). Due to conflicting data presented in the literature, a consensus cannot be reached regarding gender dominance where patients reach the secondary progressive stage of MS (Koch et al., 2008, 2010). However, it has been estimated that individuals may take a medium of 20 years from initial diagnosis to demonstrate clinically defined secondary progressive MS (Cree et al., 2021).

The established gender differences in MS risk argue that efficacy for disease modifying therapies (DMTs) may differ during the pathophysiological mechanisms driving the disease in males and females. These mechanisms may well involve the role of female sex hormones and the neuroprotective roles they convey on neuroaxonal integrity (Voskuhl et al., 2022). This is emphasized with progression in females that are postmenopausal and in men that exhibit increased brain atrophy detected upon MRI as age increases upon disease onset (Schoonheim et al., 2012a,b, 2013). Indeed, one driver of the remyelination response that can limit neurodegeneration (through the preservation of axoglial integrity) and hence disability, may well involve the neuropoietic hormone estriol (Voskuhl et al., 2016). In a study conducted by Ribbons et al. (2015) that was based on data extracted from 55 collaborating centers spread over 25 countries, it was presented that upon initial onset of MS, males manifested symptoms at an older age than females. Moreover, the recorded Extended Disability Status Score [EDSS; one of the extensively accepted clinical grading scales that assess neurological impairment and disability in MS patients based on their visual, brainstem, pyramidal, cerebellar, sensory, bowel/bladder, and cerebral function (Amiri et al., 2019)] was reported to be higher in males than that of females living with MS. Additionally, a recent study that collected data from the total population of Danish citizens with MS from 1948, described a significantly faster progression of disability in males compared to females (Ribbons et al., 2015; Magyari and Koch-Henriksen, 2022). These data suggest that the sex differences for autoimmune induction of demyelination occur earlier in women but men demonstrate more aggressive neuroaxonal injury with faster disability. The earlier onset of RRMS in females suggests that with the implementation of early DMTs, this can limit the relapse rates, delaying progression and disease burden. However, the later onset in males suggests that progressive MS demonstrates a neurobiological basis where female sex hormones play an important neuroprotective role early in life. Such in-depth epidemiological and demographic evidence highlight the need for novel therapeutic measures that target the axo-glial unit and can facilitate neuroprotection and repair of the temporal pathological lesions in the heterogeneous affected MS population. Hence, novel therapeutic development that targets neuroprotection and repair of the axo-glial unit requires the appropriate stratification of MS patients according to gender, age and the evolution of active lesions, that can all govern the responsiveness of endogenous cells (with an estrogen-rich environment), during demyelination to enhance clinical efficacy.

## 2. Pathology of multiple sclerosis

Clinical manifestations of MS depend on the severity and region of lesions occurring in the central nervous system (CNS) (i.e., brain, spinal cord, or optic nerve) (Hauser and Cree, 2020). Lesions that evolve as a hallmark of MS are caused by infiltration of peripheral immune cells (CD4/CD8+ T and B-cell lymphocytes) through a leaky blood–brain barrier that target myelin epitopes. Historically, it was assumed that MS was predominantly a T-cell mediated disease. However, a phase-II double-blind study by Hauser et al. (2008), demonstrated that B-cells play a key role during MS pathogenesis. Evidence that B-cells were involved during MS was observed in patients with RRMS who were receiving rituximab. Rituximab is a monoclonal antibody that selectively targets and depletes CD20+ B lymphocytes with demonstrated reduced relapses and total lesion load over a 24-week course, compared to patients who received placebo only (Hauser et al., 2008).

## 3. Progressive MS and evidence for molecular mechanisms leading to axonopathy

Progressive MS is clinically characterized by a steady decline in neurological function without any periods of recovery (Lublin and Reingold, 1996; Lublin et al., 2014) with the potential for eventual paralysis, loss of bowel or bladder control, and blindness (Lawthom et al., 2003; Le Scanff et al., 2008; Shi et al., 2022). There is now clear evidence that axonal damage/loss is the major determinant of profound neurological deficit in MS affected individuals (Friese et al., 2014). The molecular and cellular pathological outcomes during MS progression are characteristic hallmarks of the myelin oligodendrocyte glycoprotein (MOG)_35–55_ induced animal models of experimental autoimmune encephalomyelitis (EAE) (Mendel et al., 1995; Hasselmann et al., 2017; Kipp et al., 2017). At the stage of progressing ascending paralysis in the EAE model there are clear molecular disruptions to axonal transport [as reported by our group (Petratos et al., 2012; Theotokis et al., 2012; Lee et al., 2019)] that precede axonal dystrophy. These axonal transport deficits are also characteristic in post-mortem specimens from progressive MS patients. Moreover, innate immune activation in type-1 astrocytes and microglia are clearly demonstrated during the chronic stages of EAE and common histopathological findings in archival tissue from individuals that lived with progressive MS (Ponomarev et al., 2006; Jukkola et al., 2013).

**FIGURE 1:**
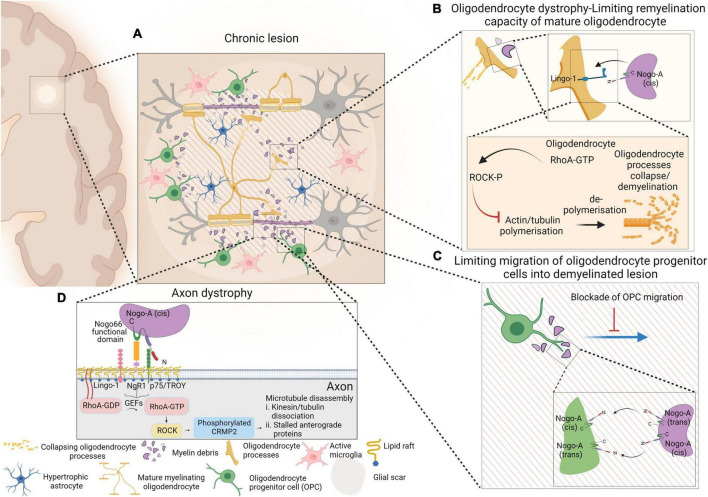
Schematic representation of events occurring within chronic lesion that lead to axonal degeneration, limiting remyelination capacity of mature oligodendrocytes and limiting migration of oligodendrocyte progenitor cells (OPCs) into the demyelinating lesion. Presence of active microglia and hypertrophic astrocytes within glial scar are depicted as well **(A)**. Despite the presence of mature myelinating oligodendrocytes within lesion environment, interaction of myelin debris with oligodendrocytes via Nogo-A-Lingo-1, leads to activation of RhoA-ROCK pathway and collapse of oligodendrocyte processes **(B)**. Nogo-A–Nogo-A interaction between OPCs and myelin debris confines OPCs within shadow plaques and blocks their migration into the glial scar **(C)**. The molecular pathway that involves in NgR1-dependent axonopathy within an inflammatory neurodegenerative milieu is also presented. Nogo-A binds to Nogo receptor-1 complex and that activates membrane-bound RhoA-GDP. Activated RhoA-GTP is released from the membrane and interacts with ROCK and that initiates phosphorylation of CRMP2. Phosphorylation of CRMP2 ultimately results in microtubular disassembly and axonal degeneration **(D)**. Figure is generated by BioRender.

Attempts have been made to correlate these pathological changes with the clinical disability EDSS score, matching neuronal loss, axonal damage, synaptic loss in demyelinating gray matter of the hippocampus, to cognitive decline (Wegner et al., 2006; Papadopoulos et al., 2009; Dutta et al., 2011). MOG_35–55_ induced EAE within a cohort of reporter mice revealed that significant reductions in inhibitory neurons along with pre-synaptic puncta observed within demyelinating gray matter throughout the hippocampus and these findings may explain the reduced spatial learning exhibited by these mice (Ziehn et al., 2010; Hammond et al., 2020). These animal experiments have led to clinicopathological investigations of cortical demyelination and ensuing neurodegeneration in MS patients (Haider et al., 2016). Significant neuronal loss and the reduction in size of neuronal somata were reported to be present within the chronic demyelinated hippocampal gray matter of progressive MS brain tissue (Papadopoulos et al., 2009). Moreover, a microarray-based gene expression study of demyelinated hippocampi revealed that there was a significant decrease in genes that are involved in axonal transport (Dutta et al., 2011). A profound deficit in anterograde axonal transport has been postulated from data generated in this study which demonstrated reduced mRNA levels of the kinesin gene family; Kinesin 1A, Kinesin 3A, Kinesin 15, Kinesin 5B, Kinesin 5C, and kinectin 1 in demyelinating hippocampi of MS patients (Dutta et al., 2011). A decreased expression of Kinesin family 1A in these lesions may suggest reduced learning enhancement/plasticity associated with hippocampal degeneration/synaptic integrity (Kondo et al., 2012). Our group, and others (Kreutzer et al., 2012; Redondo et al., 2015; Lee et al., 2019), have clearly identified axonal transport deficits through reduction of the kinesin family of axonal transport proteins in the optic nerve and spinal cords of MOG_35–55_ EAE induced mice (Lee et al., 2019; see Figure 1). These data may suggest that altered expression of kinesin and/or its dissociation from the axonal microtubule network is a major contributor to abrogated communication between neuronal networks leading to progressive neurodegeneration and cognitive decline.

## 4. Putative mechanisms by which myelin debris limit axonal regeneration and remyelination

A landmark study in 1981 (David and Aguayo, 1981) highlighted the concept that neurons could have regenerative capacity and rather than being an intrinsic feature. This capacity for repair may depend on environmental influences exerted upon axons. However, in that early study, an autologous peripheral nerve (sciatic nerve) conduit, 35 mm long, was used as a bridge between the medulla oblongata and spinal cord in a rat model. Within 22–30 weeks, it was observed that the number of axons present within the bridge originated from the medulla oblongata and spinal cord and were capable of growing up to 30 mm in length. This observation indicated that the peripheral nervous system (PNS) environment is supportive for the elongation and regeneration of axons while this does not occur within the CNS (David and Aguayo, 1981). One of the factors which contributes to integral differences between the CNS and PNS environments are the myelin producing glial cells, established early in development from their endogenous precursors (Dermitzakis et al., 2022). While the CNS myelin is produced by mature oligodendrocytes, Schwann cells are the myelin producing cells of the PNS. Among the two germinal zones within the mammalian forebrain (ventricular and sub-ventricular zones), oligodendrocytes (and astrocytes) are perinatally generated from the sub-ventricular zone (SVZ) (Levison and Goldman, 1993), while Schwann cells are generated from the neural crest (Joseph et al., 2004). A major difference between the two anatomical regions is the myelin composition. While central myelin is characterized by increased amounts of the myelin glycolipids, cerebrosides, sulfatides, and gangliosides, myelin derived from Schwann cells (Garbay et al., 2000) include the glycoproteins myelin protein zero (PO, also called MPZ) (Ishaque et al., 1980) and peripheral myelin protein-22 (Snipes et al., 1992), as major myelin membrane constituents. In 1988, a 35 and a 250 kDa membrane protein (expressed as plasma membrane protein by oligodendrocytes and myelin) was isolated from white matter within the CNS, which demonstrated potent inhibition for neurite outgrowth and fibroblast spreading (Caroni and Schwab, 1988). This finding resulted from an observation in which co-cultured sensory and sympathetic neurons with optic nerve explants allowed axon ingrowth if the 35 and 250 kDa proteins were neutralized with IN-1 and IN-2 antibodies, respectively (Caroni and Schwab, 1988).

Myelin-associated glycoprotein (MAG) (McKerracher et al., 1994), oligodendrocyte myelin glycoprotein (OMgp) (Wang et al., 2002b) and Nogo-A (GrandPré et al., 2000), classified as myelin-associated inhibitory factors (MAIFs), are among the components that exert an inhibitory effect upon neuronal outgrowth. MAG is not specific to the CNS and it is also present in myelinated peripheral nerves (Figlewicz et al., 1981). Interestingly, MAG is bifunctional, exhibiting both stimulatory and inhibitory effects toward neurite outgrowth, depending on the neuron developmental stage (Turnley and Bartlett, 1998). Turnley and Bartlett (1998), reported that embryonic spinal cord neurons cultured on a monolayer feeder of stably transfected Chinese hamster ovary (CHO) cells with MAG cDNA, were significantly elongated compared to neurons cultured without the expression of MAG. However, the same neurite outgrowth stimulatory effect was not observed with postnatal spinal cord neurons cultured in the presence of MAG transfected CHO cells (Turnley and Bartlett, 1998). Mikol et al. (1990), subsequently reported that OMgp was not expressed in Schwann cells, in PNS myelin or in any other tissue outside the CNS. However, it was later resolved that OMgp expression was not limited to myelin produced by oligodendrocytes, but were specifically expressed on the nodes of Ranvier in peripheral nerves within the PNS (Apostolski et al., 1994). Among the MAIFs, Nogo-A is specifically expressed within the myelin membrane produced by oligodendrocytes and not by Schwann cells (Fournier et al., 2001). Collectively, there is growing evidence to support the notion that Nogo-A is a major inhibitory determinant of axonal regeneration and remyelination in the CNS.

### 4.1. The integral myelin protein: Nogo-A

Nogo-A is a 1,192 amino acid, 256 kDa myelin glycoprotein localized to the CNS, capable of inhibiting axonal growth. The interaction between Nogo-A and WW domain-containing E3 ubiquitin protein ligase 1 (also called TGIF interacting ubiquitin ligase 1 and Atropine-1-interacting protein 5), suggests that ubiquitination plays a critical role in breakdown and turnover of Nogo-A. In eukaryotic organisms, the reticulon (RTN) family of proteins is a predominant member of the membrane-associated protein group, which includes Nogo-A. Structurally, Nogo-A contains a conserved C-terminal region consisting of two hydrophobic regions flanked by a hydrophilic loop. There are four types of RTNs expressed in mammalian cells – RTN4 is known as Nogo. Despite the presence of some RTNs on the cell surface, the di-lysin motif in the C-terminus of RTNs inclusive of Nogo, causes the proteins to mainly localize on the endoplasmic reticulum in cells (Bongiorno and Petratos, 2010). The three isoforms of Nogo (isoform A, B, and C) arise due to alternative splicing and the promotor sequence of the *RTN4* gene (Huber et al., 2002). Similarly, these isoforms share a 188 amino acid carboxyl-terminus, consisting of two hydrophobic domains that encompass a hydrophilic 66 amino acid loop (Bongiorno and Petratos, 2010). When Nogo (isoform A) is expressed on the cell surface, its three active sites, including Nogo-66, Nogo-A (800 amino acids) and the N-terminus are believed to be extracellular. Principally, Nogo-A and its Nogo-66 domain function to restrict neurite growth and promote growth cone collapse, whereas, the N-terminus of the Nogo isoform A can either directly or indirectly limit axonal growth via the inhibition of integrin signaling at the growth cone (Hu and Strittmatter, 2008).

As mentioned previously, axonal regeneration can be achieved in the adult mammalian PNS, however the same regeneration cannot be efficiently achieved in the CNS. Despite many classes of axons in the CNS that exhibit an inherent capacity to extend long distances in PNS grafts, axonal growth capabilities are compromised due to the inhibitory nature of CNS white matter (David and Aguayo, 1981). Nogo-A is expressed by CNS oligodendrocytes but not by PNS Schwann cells (Fournier et al., 2001). Nogo-66 is expressed at the extracellular surface and at the endoplasmic reticulum lumen of transfected cells (GrandPré et al., 2000), in the Golgi complex (Oertle et al., 2003), and in the innermost lamella of the myelin sheath produced by oligodendrocytes (Fournier et al., 2001). Nogo-66 exerts its inhibitory effects on axons and does not cause any alteration in non-neuronal cell morphologies, however, the N-terminus of Nogo-A has the ability to alter the morphology of neuronal or non-neuronal originating cells (Fournier et al., 2001). These data argue that amino-Nogo-A can signal cytoskeletal alterations in neurons.

A recently discovered function of Nogo-A is its regulatory effects on synaptic plasticity within the uninjured adult CNS (Zemmar et al., 2018). Nogo-A is present in both oligodendrocytes (Kuhlmann et al., 2007; Zemmar et al., 2018) and in surrounding oligodendrocyte processes on myelinated axons (Wang et al., 2002c). However, the highest expression of Nogo-A is reported to be on the inner and outer myelin sheaths and on oligodendrocyte cell bodies (Buss et al., 2005). In a detailed proteomic study (Buss et al., 2005), it was revealed that Nogo-A was mainly expressed in oligodendrocytes, motor neurons and interneurons within the spinal cord, in sensory neurons within the dorsal root ganglia, in oligodendrocytes within the cerebral cortex, hippocampus, and cerebellum, in oligodendrocytes of the red nucleus and inferior olive within the brain stem, and in retinal ganglion cells and inner and outer nuclear layers within the retina. Moreover, low expression of Nogo-A was demonstrated in layers II and III, V and VI within the cerebral cortex, in CA 1–4 within the hippocampus, in the granule cell layer and molecular layer within the cerebellum. Astrocytes, microglia, satellite cells, Schwann cells, fascia dentata, Purkinje cells, deep cerebellar nuclei, and substantia nigra are reported to be among the cells that do not express Nogo-A (Buss et al., 2005). Aiming to establish which form of Nogo-A was responsible for regulating synaptic plasticity, it was determined that neuronal Nogo-A regulated proximal dendrites, whereas oligodendrocytic Nogo-A regulated distal dendrites (Zemmar et al., 2018), highlighting the divers structural and physiological mechanisms that Nogo-A may govern in the CNS.

### 4.2. How does the putative ligand for NgR1, Nogo-A, exert its potent cellular inhibitory effects?

Myelin debris that exists extracellularly express myelin associated inhibitory factors which can: (a) limit myelinogenic potential of oligodendrocytes within the lesion site and so denuded axons generated as a result of limited remyelination are more prone to degeneration; and (b) inhibit axonal regeneration via Nogo receptor 1 (NgR1)-dependent signaling to impair the axonal transport machinery (Chong et al., 2012; Petratos et al., 2012; Theotokis et al., 2016; Lee et al., 2019).

The inhibitory effect on axonal growth by Nogo-A is exerted via its high affinity interaction with its receptor Nogo-66 (NgR). NgR is a 473 amino acid glycosylphosphatidylinositol-linked protein (GPI-linked protein), consisting of a signal sequence followed by eight leucine-rich-repeat (LRR) domains, cysteine rich at its carboxyl terminus (a unique region), and a GPI anchorage site. Consistent with the function of NgR in limiting axonal regeneration and adult CNS plasticity, the expression of NgR mRNA in its full length was only detected in brain and at lower levels in the heart and kidneys but absent from other peripheral tissue. Even though NgR is expressed throughout the brain, its highest expression is recorded in gray matter (Fournier et al., 2001). The amyloid precursor protein (Zhou et al., 2011), MAG (Robak et al., 2009), OMgp (Wang et al., 2002b; Huang et al., 2012), and the cleaved forms of β-amyloid protein (Aβ40 and Aβ42) (Zhao et al., 2017) have been identified as NgR ligands. It is worth mentioning here that among the NgR ligands, Nogo-A has more potent neurite outgrowth effects than the other MAIF ligands MAG and OMgp (Cafferty et al., 2010).

Interestingly, the cognate ligands of NgR (OMgp, MAG, and Nogo-A) share no similarity in sequences or domains but all exert an inhibitory effect on axonal outgrowth via a competitive interaction with the NgR-specific co-receptors that multimerize in the lipid rafts to promulgate signal transduction, since NgR *per se* is incapable of transducing transmembrane signals by itself. NgR is a GPI-linked protein (Fournier et al., 2001) which attaches to the outer surface of plasma membranes; it does not span the membrane and due to the hydrophobic nature of its phospholipid tail, it does not directly interact with intracellular cytoplasmic and cytoskeletal components (Robinson, 1997). The pleiotropic neurotrophin receptor P75 (p75^NTR^), leucine-rich repeat and immunoglobulin-like domain-containing Nogo receptor-interacting protein 1 (LINGO-1) and TROY (member of tumor necrosis factor receptor family) are other known transmembrane co-receptors of MAIFs that function as co-receptors to NgR (for review see Lee and Petratos, 2013; Theotokis and Grigoriadis, 2018). Yamashita et al. (2002), initially reported that treating adult wild type mice with a soluble chimeric form of MAG, inhibited neurite outgrowth of dorsal root ganglion (DRG) and postnatal cerebellar neurons, however the same neurite outgrowth inhibitory effect of MAG was not detected in mice carrying an exon III mutation in the *p75* gene. Confocal microscopy imaging revealed the colocalization of MAG and p75^NTR^ on DRG neurons of wild type mice, but remarkably, binding of MAG to DRG neurons was also detected in *p75*-null mutant mice. These findings indicate that p75^NTR^ cannot be the binding partner to MAG. Furthermore, in the initiation of neurite outgrowth inhibitory mechanisms by MAG, p75^NTR^ plays a cooperative role or rather acts as an intermediary co-receptor (Yamashita et al., 2002). However, it was discovered by Wang et al. (2002a) that NgR engages a multimeric complex that includes p75^NTR^ in CNS neurons to mediate the MAG-dependent neurite inhibitory effect. Using rat postnatal cerebellar granule neurons in culture, it was reported that p75^NTR^ can immunoprecipitate together with NgR proteins and incorporation of the soluble MAG protein enhanced the formation of the NgR-p75^NTR^ multimeric complex while blocking interaction of NgR and p75^NTR^, reducing neurite outgrowth inhibition. It was also demonstrated that by truncating the intracellular domain of p75^NTR^, neurite outgrowth was achieved in the presence of MAIFs. The results collectively confirmed that p75^NTR^ is the co-receptor to NgR and it mediates the downstream signaling pathway by transducing signals which occur following ligand binding to NgR (Wang et al., 2002a).

Given that neurite outgrowth inhibitory effects of MAIFs and their interactions with the NgR-p75^NTR^ complex is mediated by the small GTPase RhoA (Ras homolog gene family, member A)-dependent pathway activation (Stern et al., 2021) (later discussed in more detail), it was reported by Mi et al. (2004) that in cerebellar granular neurons, OMgp is also able to inhibit neurite outgrowth in a similar manner. However, the same inhibitory effect of OMgp was not detected in non-neuronal COS-7 cells which were transfected with a combination of NgR1 and p75^NTR^. This observation indicated that in neuronal cells, there might be other possible receptors activated within the NgR1-p75^NTR^ complex, dependent on the ligand and receptors being expressed. In that study, LINGO-1, a transmembrane protein which has the Nogo receptor interacting domains (LRR and an Ig domain) and can associate with p75^NTR^ clustered in lipid raft signaling domains of neural cell membranes, was identified as the third co-receptor. LINGO-1 was found to be highly expressed in the cerebral cortex in contrast to its low expression within the spinal cord, while there was no LINGO-1 detected in non-neuronal tissues. In a series of conformational experiments (Mi et al., 2004), it was discovered that COS-7 cells transfected with NgR1 and LINGO-1 were still unable to initiate RhoA-GTP (guanosine triphosphate) activation in the presence of exogenously added OMgp. Confocal microscopic imaging determined the colocalization of NgR1, p75^NTR^ and LINGO-1 were all expressed on DRG neurons and as opposed to COS-7 cells transfected with NgR1 and LINGO-1, COS-7 cells transfected with NgR1-p75^NTR^-LINGO-1 were able to initiate the downstream RhoA signaling pathway. Therefore, LINGO-1, was reported as the co-receptor to the NgR1-p75^NTR^ signaling as a multimeric complex (Mi et al., 2004). Results reported by a number of studies (Park et al., 2005; Bracken and Turrigiano, 2009; Baho et al., 2019) raise the notion that there should be a functional homolog to p75^NTR^ in mediating myelin inhibition. As opposed to NgR (Wang et al., 2002c) and LINGO-1 (Mi et al., 2004), which are widely expressed in the CNS, p75^NTR^ expression is selective, as it is expressed at embryonic stages of CNS development (Meier et al., 2019) with its expression downregulated postnatally and restricted to small subpopulations of stem cells (0.3%) within the SVZ, which generate neurons during adulthood (Young et al., 2007). Among adult neuronal cell populations, expression of p75^NTR^ can be detected in DRG (Koike et al., 2019) and on basal forebrain neurons (Xie et al., 2019), therefore to compensate for the restricted expression of p75^NTR^, it was anticipated that there exists a functional homolog to p75^NTR^. A study conducted in 2005 (Park et al., 2005) reported that when DRG neurons were exposed to Nogo-A or OMgp, almost all the exposed cells exhibited inhibitory neurite outgrowth effects, while only 60–70% of DRG neurons were positive to p75^NTR^. Therefore, several tumor necrosis factor (TNF) receptor family candidates (of which p75^NTR^ belongs), including TROY, death receptor 6 and TNFR1A (TNF receptor superfamily member 1A), were examined as a potential homolog to p75^NTR^. It was found that TROY which is highly expressed in most of the CNS as well as DRG neurons could bind to both NgR and LINGO-1 to form a receptor complex and the exposure to exogenous MAIFs could confer RhoA activation within COS-7 cells, co-transfected with NgR, LINGO-1 and TROY. Hence, TROY was identified as the mediating coreceptor to the NgR multimeric signaling complex (Park et al., 2005), demonstrating the variability in MAIF-dependent signaling on different neuronal populations.

### 4.3. NgR1-dependent axonopathy

A canonical pathway known to mediate the growth inhibitory effects on neurons is RhoA, as an intracellular regulator of GTPase in the cytoskeleton. The pathfinding and formation of axons are regulated with Rho family members including RhoA, Rac1, and Cdc42. This regulation acts through cyclic activation (GTP-bound state) and inactivation (GDP-bound state) of intracellular Rho proteins, anchored to the inner leaflet of the plasma membrane of lipid rafts that become released upon their activation through phosphorylation (for review see Garcia-Mata et al., 2011; El Masri and Delon, 2021). The downstream target following Rho activation is p160ROCK (Rho-associated coiled-coil containing kinase), a member of the serine/threonine kinase family. Two isoforms of p160ROCK exist, ROCKI and ROCK II, with the latter being highly expressed within the brain compared to ROCKI (Nakagawa et al., 1996). The contribution of activated Rho (GTP-bound Rho) and its downstream target p160ROCK in determining axonal outgrowth in mammalian CNS neurons was first reported by Bito et al. (2000). In this study, cerebellar granule neurons were transfected with a dominant active form of RhoA. The numbers of neuronal processes (neurites) had decreased while inhibition of RhoA, by the C3 enzyme, resulted in an increased number of neurites. Interestingly, when p160ROCK, downstream of GTP-RhoA, was inhibited by the small ROCK inhibitor molecule Y-27632, in cerebellar granule neurons which were already transfected with a dominant active form of RhoA, the expected neuronal growth deficit was neutralized. While it could not be concluded that there was a direct relationship between the Rho/ROCK pathway and axon length, it was evident that the Rho/ROCK pathway controlled the size of the neuronal growth cone at the tip of axons with the rationale being that overexpression of RhoA significantly reduced the size of the growth cone while inhibition of RhoA or ROCK lead to dramatic augmentation of growth cones (Bito et al., 2000). By using the MOG_35–55_ EAE mouse model of MS-like disease, we previously have reported that by overexpressing the microtubule associated phosphoprotein, collapsin response mediator protein 2 (CRMP-2) with a site-directed mutagenesis to the ROCKII. Threonine 55 phosphorylation site, the neuroinflammatory induced axonopathy could be abrogated (Petratos et al., 2012; Lee et al., 2019). This was a series of landmark experiments since high levels of pThr555-CRMP-2 were detected within spinal cord axons of EAE-induced mice around inflammatory demyelinating lesions. Moreover, similar expression pattern was also observed within archival MS brain and spinal cord tissue. The link between NgR1-dependent signaling with downstream pThr555-CRMP-2 was identified initially in mice mutant for the *ngr1* allele (*ngr1^–/–^)*, where the level of pCRMP-2(Thr555) detected in axons near inflammatory lesions was significantly reduced, with abrogated axonal degeneration observed. This was then targeted in therapeutic intervention experiments whereby, passive immunization of EAE-induced mice with anti-Nogo-A antibody also resulted in reduced pCRMP-2(Thr555) detection in the spinal cord, with maintained axonal integrity and abrogated clinical progression. Hence the reported data collectively confirms the contribution of CRMP-2 in its phosphorylated form, in axonal degeneration initiated by NgR1-dependent axonopathy (Petratos et al., 2012).

It is well established that CRMP-2 plays a central role in regulating growth cone development within the unphosphorylated form and axon elongation via regulation of microtubule assembly and Numb-mediated endocytosis (Arimura et al., 2005). Growth cones are made up of microtubules and neurofilaments at the center and actin microfilaments at the edge. Establishment of the microtubular array is facilitated by transport of tubulin heterodimers (α- and β-tubulins) and tubulin polymerization at the positive-ends of microtubules, the mechanisms which ultimately result in microtubular assembly and axonal growth. CRMP-2 contributes to microtubular assembly and to axonal growth by functioning as a carrier of tubulin heterodimers to the positive-end of microtubules, followed by eventual co-polymerization for the growth of microtubules (Fukata et al., 2002). Another mechanism by which CRMP-2 facilitates axonal growth is via its interaction with Numb, a protein responsible for endocytosis and recycling of L1 (Nishimura et al., 2003), a neuronal cell-adhesion molecule that plays a role in regulation of axonal outgrowth and axonal guidance (Chaisuksunt et al., 2000; Guseva et al., 2018) at the growth cone. Phosphorylation of CRMP-2 by ROCK, can inhibit the ability of CRMP-2 to bind with tubulin heterodimers and Numb, leading to the inhibition of axonal growth and disruption of assembled-microtubule maintenance (Arimura et al., 2005). In contrast, inhibiting axonal growth interrupts the formation and maintenance of the synaptic connection between neurons and this alone can lead to degeneration/death of presynaptic neurons (Zhang and Benson, 2001; Meng et al., 2015). Hence, the NgR1-dependent phosphorylation of CRMP-2 can limit the growth of axonal microtubules and contribute to promulgation of neurodegenerative processes with ongoing inflammatory demyelination during MS.

### 4.4. Nogo-A inhibition of remyelination by limiting migration and maturation of oligodendrocyte progenitor cells

During demyelinating diseases such as MS, myelin sheaths are targeted for destruction by the immune system. Despite the presence of oligodendrocyte progenitor cells (OPCs) and myelin-forming mature oligodendrocytes throughout the lesion environment, the replacement of myelin does not occur efficiently enough to fulfill the requirement set by demyelinated axons (Wolswijk, 2000). Detection of NG2 (neuron-glial antigen 2)-positive OPCs in MS lesions was first reported by Chang et al. (2000) establishing that NG2 is an integral membrane proteoglycan only expressed on OPCs but not on oligodendrocytes (Reynolds and Hardy, 1997; Dawson et al., 2003). According to Chang et al. (2000), NG2-positive OPCs are present in chronic MS lesions exhibiting elongated phenotypes which are phenotypically similar in appearance in the normal human adult brain. However, the density of NG2-positive cells was reduced compared to healthy brains which could be explained by death or removal of existing NG2-positive OPCs from lesion sites. Furthermore, NG2-positive OPCs were detected in acute MS lesions, not within the demyelinated area but at the borders of lesions (Chang et al., 2000). In contrast, it was reported that in normal-appearing white matter, a subpopulation of NG2-positive OPCs within lesions expressed the p75^NTR^ receptor, a marker which was previously reported to be associated with cell-signaling and death (Chang et al., 2000, 2002; Petratos et al., 2003). Additionally, in a study conducted by Romanelli et al. (2016) the presence of oligodendrocytes was present in actively demyelinating MS lesions (Rodriguez et al., 2014; Romanelli et al., 2016). This study demonstrated that in MS patients and in the EAE animal model, oligodendrocyte damage was initiated by immune attack to the myelin and not the oligodendrocyte soma, indicating that the centripetal-orientated model of damage spread outside-in, from myelin to the soma. This eventuates in myelin degeneration due to myelinosome formation (local myelin out-folding). Interestingly, the presence of myelinosomes were also detected on normal appearing axons both in MS lesions and in the EAE model, indicating that initiation of myelin damage in neuroinflammatory lesions may be independent of axonal damage (Romanelli et al., 2016).

Since it is evident that viable oligodendrocytes and OPCs exist within MS lesions, it is important to understand which underlying mechanisms may control the inherent ability of oligodendrocytes to myelinate axons under neuroinflammatory conditions. Evidence demonstrates that the same mechanisms which regulate axonal growth and regeneration (NgR1 and Lingo-1 signaling), may also regulate myelin sheath formation by oligodendrocytes (Baer et al., 2009). Baer et al. (2009), determined that soluble myelin molecules inhibit differentiation of OPCs which is mediated via activation of the RhoA-ROCK pathway, while inhibition of ROCKII which is expressed by OPCs (downstream mediator to RhoA) promotes OPC differentiation in the presence of myelin. However, in that study, as opposed to Lingo-1, expression of NgR was not detected in OPCs and the precise component within myelin which inhibits OPC differentiation remains unknown (Baer et al., 2009). Similarly, Kotter et al. (2006), demonstrated that myelin debris from demyelination in focal lesions within the rat brainstem, could inhibit differentiation of OPCs, but not the recruitment of OPCs to the lesion site (Kotter et al., 2006). In order to identify particular components within myelin debris that may be inhibitory to oligodendrocytes, Chong et al. (2012), coated polystyrene beads with a high culture density of OPCs co-cultured with DRG neurons. They reported that oligodendrocyte membranes exhibited a twofold decrease in quantity, together with the length of myelin internodes compared to controls (beads coated with membrane of other cell types, i.e., astrocytes). This study was extended by including myelin components in the co-cultures. A particular component was the amino-terminal of Nogo-A (Nogo-Δ20), which was identified as an inhibitory factor to myelin formation and in a conformational analysis, it was also shown that knocking down of Nogo-A expression in OPCs before being used for coating the beads, significantly increased the myelinogenic potential of oligodendrocytes.

However, in contrast to Baer et al. (2009), Kotter et al. (2006), and Zhao et al. (2007), no association between oligodendrocyte, myelin debris and OPC differentiation was determined (Chong et al., 2012). Two inhibitory domains of Nogo-A exist: Nogo-66 which binds to NgR-1; and Nogo-Δ20, which was identified as the region of Nogo-A that contributes to inhibiting the myelinogenic potential of oligodendrocytes, consistent with the reports by Baer et al. (2009). The expression of LINGO-1 exists in axons and oligodendrocytes, and higher levels of expression in stimulated OPCs may have inhibitory roles in axonal regeneration and growth or as a factor to negatively regulate oligodendrocyte differentiation and myelination. It was reported that knocking down LINGO-1 in OPCs, resulted in the formation of highly differentiated mature oligodendrocytes with abundant myelin sheath structures, while overexpression of LINGO-1, inhibited differentiation of OPCs and myelination. Due to the role that RhoA-GTP has on OPC differentiation (Liang et al., 2004), it was postulated that LINGO-1 exerts an inhibitory effect via the intracellular upregulation of RhoA-activity (Mi et al., 2005; Zhao et al., 2007). Despite other groups reporting the same inhibitory effect of LINGO-1 on oligodendrocyte myelinogenesis (Mi et al., 2008), the activation of LINGO-1 signaling remains elusive. A schematic representation of events occurring within chronic lesion that lead to Nogo-A inhibition of remyelination by limiting migration and maturation of oligodendrocyte progenitor cells, along with NgR1-dependent axonopathy are represented in Figure 1.

## 5. Biological therapies targeting Nogo-A/NgR1

The inhibitory effect of Nogo-A on myelination and OPC recruitment to lesion sites, could potentially be a trigger to pathological events that govern axonal degeneration, and so antagonizing Nogo-A function in the pathological tissue milieu, may be of therapeutic interest for MS. A recent study conducted by Ineichen et al. (2017), successfully demonstrated that function-blockade of Nogo-A with a monoclonal anti-Nogo-A antibody had improved the recovery of fine motor control in animals as a result of significantly enhanced remyelination/myelin repair and axonal sprouting throughout gray matter regions. Anti-Nogo-A antibody treatment was administered to these animals intrathecally for 14–16 days and as result, animals recovered significantly compared to control animals (Ineichen et al., 2017). Of note, it was previously demonstrated that the interaction of NgR1 and newly synthesized myelin, in the context of PNS inflammation, mediated efflux of macrophages from the sites of infiltration, resulting in resolution of the inflammatory response after injury (Fry et al., 2007). However, anti-Nogo-A antibody treatment showed no such modulatory effects on the efflux of macrophages/microglia from inflammatory lesions within the CNS and had no effect on migration, proliferation or differentiation of OPCs, suggesting that enhanced remyelination/myelin repair as a result of anti-Nogo-A antibody treatment may be due to an increased myelinogenic potential of oligodendrocytes, as defined by an increased number of internodes formed by a single oligodendrocyte. Collectively, interventional studies following anti-Nogo-A antibody administration have highlighted the promise of antagonizing Nogo-A for the treatment of RRMS. This would be of particular interest in treating the chronic-progressive stages of MS, where there exists a heightened and deleterious macrophage/microglia activation, and for which currently no effective therapies are available (Chong et al., 2012; Ineichen et al., 2017).

### 5.1. Development of NgR1-specific targeted therapies

Traumatic damage to the mammalian spinal cord is a condition which severely compromises locomotor ability and often the symptoms manifest at the level of spinal cord injury (SCI) due to disruption of neuronal networks between the brain and spinal cord. Upon injuries to the CNS, the ability of axonal regeneration is compromised. This mainly occurs due to inhibitory effects of MAIFs, of which Nogo-A has been demonstrated as the most potent throughout the injury milieu, by which NgR1 signaling limits axonal growth (Hu and Strittmatter, 2008; for review see Lee and Petratos, 2013). In a sophisticated study conducted by GrandPré et al. (2002), the identification of residues on Nogo-66 which contribute to the high-affinity binding to its cognate receptor NgR established that the N-terminus of Nogo-66 with amino acid residues 30–33 region is required. The Nogo-66 (1–40) peptide (Nogo extracellular peptide, residues 1–40, NEP1-40) was then introduced as a potent inhibitor that blocked Nogo-66-dependent axon growth inhibition (GrandPré et al., 2002). Studies by Fournier et al. (2002) contributed to the development of a soluble, truncated NgR containing amino acids 1–310 of Rat NgR (NgR(310)ecto) that could bind to Nogo-66 and antagonize Nogo-A (Fournier et al., 2002). Further studies aimed to stabilize and promote the purification efficiency and the soluble NgR(310)ecto was fused with the rat IgG Fc domain (NgR(310)ecto-Fc). Moreover, it was sustained that as opposed to NEP1-40, which could only block Nogo-66, NgR(310)ecto-Fc could effectively bind to all three ligands of NgR1 (Nogo-66, MAG, and OMgp) and hence effectively block NgR1-specific activation (Li et al., 2004). In an attempt to optimize the NgR(310)ecto-Fc, incorporation of alanine to replace Cys^266^ and Cys^309^ was demonstrated to eliminate disulfide scrambling and to reduce the mis-linked and heterogeneous disulfide pattern (Weinreb et al., 2010). The AXER-204 drug, as a human Nogo trap fusion protein, is a Human version of NgR1(310)ecto-Fc, which has been developed by ReNetX Bio, Inc., and was reported in a completed phase I/II clinical trial investigating chronic SCI (NCT03989440). However, the safety, efficacy, and toxicological profile of such reagents on non-human primate SCI models were not widely studied. To address this issue and to further provide support for the clinical development of NgR1(310)ecto-Fc, a study was conducted on the African green monkey (Wang et al., 2020). For the intrathecal toxicology study, 30 mg/day (14-day range, in female and male monkey) and 20 mg/every other day (104-day and 108-day in female and male monkey, respectively) of NgR1(310)ecto-Fc [prepared as 10 mg/ml in phosphate buffered saline (PBS)] was given to the test groups, followed by 5 weeks of recovery. The dosage given to animals was approximately 10 times higher than that previously reported (Wang et al., 2014). Toxicology evaluation was conducted according to the parameters of body weight and condition scoring, food consumption, clinical observation, mortality, ophthalmic observation, ECG measurement, measure of blood pressure, body temperature, rate of heart and respiration and anatomical pathology. For *in vivo* studies, C5/C6 right hemisection was performed on animals under anesthesia. After inducing the injury, behavioral analysis of animals was performed by scoring on a point system in which normal behavior is scored as zero and the severe bilateral neurological impairment scored 54 as the maximum score. One month after inducing SCI, an L3–4 laminectomy was performed on animals under anesthesia and via a secured intrathecal catheter, 2 ml of NgR1(310)-Fc solution (20 mg human NgR1(310)-Fc protein equivalent to 0.10–0.17 mg/kg/day) was administered to test animals, for the entire 4-months. A vehicle solution (PBS) was used on the control animals. The behavioral analysis showed that in the vehicle control group, the left hindlimb was used 4% of the time for feeding, which indicated a strong disuse of the right forelimb as a consequence to the SCI. In contrast, in the treatment group, animals preferred to use their affected right forelimb by 17% which was higher than that of control (6%). Similarly, during walking and climbing, the vehicle group was using their hindlimb significantly less efficiently than the treatment group. In contrast to control animals, the digit score of animals in the treatment group improved significantly over time. The behavioral improvement of treatment animals that underwent treatment, the lesion size and fibrotic scar architecture throughout the spinal cord was compared between the two groups and no significant difference was found. The results indicated that NgR1(310)ecto-Fc does not alter chronic scaring, lesion size and inflammatory status of SCI (Wang et al., 2020). Even though there was no difference between the orientation and pattern of CST fibers, there were significant increases in the abundance of CST fibers in the treatment group as compared to controls. In comparison to the control groups, the gray matter axon length was reported to be significantly increased in the treatment groups in both the lesion and intact sides of the spinal cord. The finding of this study demonstrated that NgR1(310)ecto-Fc could only promote axonal growth and does not alter scarring, lesion size or microglial response. This was an expected observation and was consistent with previous reports, as NgR1(310)ecto-Fc is designed to block the activity of Nogo, MAG, and OMgp – the main contributors of axonal growth inhibition (Wang et al., 2020).

### 5.2. How could the cellular delivery of biologicals, designed to antagonize myelin protein inhibition to repair in the CNS, be translated?

**FIGURE 2:**
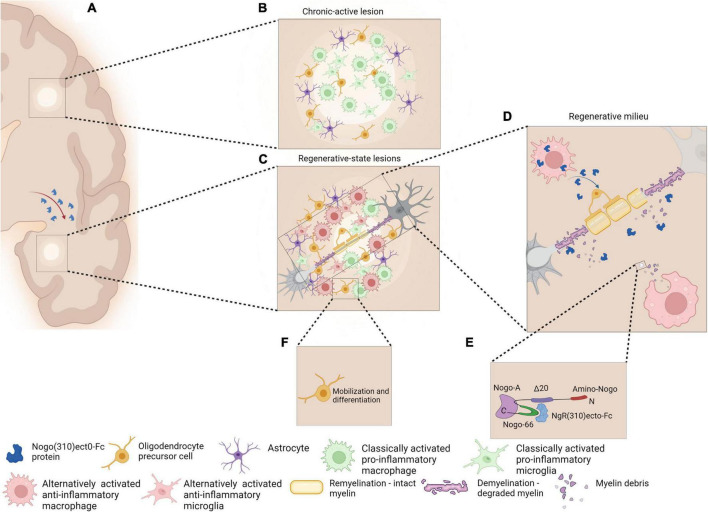
Schematic representation of chronic active MS lesions upon cellular delivery of the therapeutic protein, NgR(310)ecto-Fc **(A)**. Chronic active lesions in MS white matter are characterized by the presence of classically activated pro-inflammatory phenotype microglia/macrophage with occurrence of partial remyelination is observed as shadow plaques **(B)**. Regenerative milieu through MS plaques may be possible as a result of a novel cellular delivery of NgR(310)ecto-Fc protein to the site of inflammatory lesions. The modification of the diseased tissue milieu may shift the phenotype of microglia/macrophages from a classically active pro-inflammatory to an alternatively activated anti-inflammatory phenotype **(C)**. Anti-inflammatory microglia/macrophages may increase phagocytosis of NgR(310)ecto-Fc-bound myelin debris to expedite neural repair **(D,E)**. The events favor differentiation and mobilization of OPCs to the site of lesions and the enhancement of remyelination **(F)**. Figure is generated by BioRender.

The cellular therapeutic approach is the best possible delivery system that is translatable. A schematic presentation of events occurring upon cellular delivery of NgR(310)ecto-Fc is presented in Figure 2.

The therapeutic effect of direct delivery of NgR(ecto)-Fc is being addressed by our group since all aspects of CNS neuropathology and the clinical manifestations occur in the lentiviral-transduced hematopoietic stem cell (HSC)-transplanted recipient mice following EAE, either with or without NgR(ecto)-Fc (Ye et al., 2023). The therapeutic effects in the transplanted mice occur following the peak of EAE where neurobiological recovery is observed. The principle of directly targeting inflammatory demyelinating lesions without potential off-target effects is fundamental to this directed therapeutic approach, superior to other strategies where ubiquitous targets may affect neural circuits. This is highlighted by our very recent findings that demonstrate profound modification of the spinal cord lesions where neurorepair is exhibited in the recipient mice (Ye et al., 2023). Since Nogo-A has been an attractive target in neurological research and recently, a clinical phase I safety, tolerability and pharmacokinetics trial for RRMS has been completed (NCT01435993), it is fundamentally important to ensure that the disease-associated lesional milieu is modified to promote repair and the best chance for neurophysiological recovery. This specific clinical trial utilized a therapeutic monoclonal antibody targeting the N-terminus of Nogo-A designated as ozanezumab by the sponsor (GlaxoSmithKline) and was abruptly terminated due to questions pertaining to preclinical model data exclusion (ClinicalTrials.gov, 2020).^1^ A phase II study has also been completed with ozanezumab for amyotrophic lateral sclerosis, reporting no efficacy in the drug treatment in a double-blind controlled trial (Meininger et al., 2017). Despite termination of the previous trial due to serious data reporting errors (ClinicalTrials.gov, 2020; see text footnote 1), targeting myelin ligands can be effective in specific neurological conditions with an appropriate pre-clinical design and clearly defined therapeutic window. Rationale of the current approach for future clinical research has now been articulated in our recent study whereby our experiments have clearly identified modulation of disease-associated macrophages/microglia and A1 astrocyte neurodegenerative phenotypes to alternative-activated neuroreparative phenotypes respectively, during the targeted cellular delivery of the NgR-Fc therapeutic protein. This would be of great benefit potentially to progressive MS patients that demonstrate smoldering lesions that exhibit specific activity of these phenotypes expanding the neurodegenerative outcomes in the CNS (Ye et al., 2023).

Older inactive lesions are paucicellular and consist of hypertrophic astrocytes. Evidence exists that these astrocytes limit the spread of inflammatory milieu silencing active lesions (Rao et al., 2019). The key to these lesions throughout the CNS is to promote more A2-astrocytes near these lesions and potentiate the production of neurotrophic and anti-inflammatory mediators to support nearby myelinated fibers so that their integrity is sustained and further progression may be halted. In regard to inactive lesions that do not demonstrate myelin debris but are enriched within the glial scar, this is the most challenging question that researchers have been grappling with for many years. While it is true that the ectodomain of NgR can bind to chondroitin sulfate proteoglycans, a major component of the glial scar, there is no model of MS that replicates this chronic-inactive lesion phenotype. However, our work has uncovered that the targeted delivery of NgR-Fc to active demyelinating lesions through the transplantation of HSCs during EAE, modifies the astrocyte profile profoundly toward a reparative A2 phenotype and we are currently investigating the molecular mechanism that drives this reparative profile. Whether this would have direct implications to inactive MS lesions is uncertain but a recent study by Stephen Strittmatter’s group at Yale School of Medicine successfully demonstrated that glial scar formation was significantly reduced in a chronic non-human primate model of SCI utilizing the daily delivery of the NgR1(310)-Fc (AXER-204) (Wang et al., 2020), currently an active clinical phase I trial in chronic SCI (ClinicalTrials.gov, NCT03989440).

## 6. Conclusion

Multiple sclerosis demonstrates neurological progression during profound alterations to the CNS parenchyma with reactive changes promoting neuroaxonal dystrophy. The pleotropic signaling mechanisms that are orchestrated through NgR1, destabilize neural cell cytoskeletal integrity that manifests throughout chronic-active lesions as neurophysiological deficits and eventual loss of tissue architecture. By therapeutically targeting MAIFs at neuropathological lesion sites, during chronic-active inflammation may provide salient neuroprotective measures to limit the burden of disease during MS over time. Novel therapeutic approaches are currently being devised to deliver antagonists to the lesion-associated MAIFs in an attempt to halt the promulgation of neurodegenerative changes exhibited during MS progression. These approaches may include the cellular delivery of NgR-Fc to *trap* Nogo-A and clear it from the diseased tissue milieu, thereby promoting an environment of neurorepair. Such novel therapeutic interventions may limit MS progression or indeed promote neurorepair and neurological recovery of individuals living with the disease.

## Author contributions

ZR wrote the manuscript and generated the figure. SP, EO, MP, PT, and NG reviewed and edited the manuscript. SP generated the conceptual framework of the manuscript. All authors had read and agreed to the published version of the manuscript.

## Footnotes

1
https://ClinicalTrials.gov/show/NCT01435993
